# Inhibition of lectin-like oxidized low-density lipoprotein receptor-1 reduces leukocyte adhesion within the intestinal microcirculation in experimental endotoxemia in rats

**DOI:** 10.1186/cc9367

**Published:** 2010-12-10

**Authors:** Martin Landsberger, Juan Zhou, Sebastian Wilk, Corinna Thaumüller, Dragan Pavlovic, Marion Otto, Sara Whynot, Orlando Hung, Michael F Murphy, Vladimir Cerny, Stephan B Felix, Christian Lehmann

**Affiliations:** 1 Department of Internal Medicine B, University Hospital Greifswald, Friedrich-Loeffler-Strasse 23 a, D-17475 Greifswald, Germany; 2 Research Center of Pharmacology and Experimental Therapeutics, University Hospital Greifswald, Friedrich-Loeffler-Strasse 23 d, D-17475 Greifswald, Germany; 3 Department of Anesthesia, Dalhousie University, 1276 South Park St., Halifax, NS, B3 H 2Y9, Canada; 4 Department of Anesthesiology and Intensive Care Medicine, University Hospital Greifswald, Friedrich-Loeffler-Strasse 23a, D-17475 Greifswald, Germany; 5 Department of Anesthesiology and Intensive Care Medicine, University Hospital Hradec Kralove, Charles University in Prague, Sokolska 581, 500 05 Hradec Kralove, Czech Republic

## Abstract

**Introduction:**

Lectin-like oxidized low-density lipoprotein receptor-1 (LOX-1), the major endothelial receptor for oxidized low-density lipoprotein, is also involved in leukocyte recruitment. Systemic leukocyte activation in sepsis represents a crucial factor in the impairment of the microcirculation of different tissues, causing multiple organ failure and subsequently death. The aim of our experimental study was to evaluate the effects of LOX-1 inhibition on the endotoxin-induced leukocyte adherence and capillary perfusion within the intestinal microcirculation by using intravital microscopy (IVM).

**Methods:**

We used 40 male Lewis rats for the experiments. Ten placebo-treated animals served as a control. Thirty animals received 5 mg/kg lipopolysaccharide (LPS) intravenously. Ten endotoxemic rats remained untreated. In 10 LPS animals, we administered additionally 10 mg/kg LOX-1 antibodies. Ten further LPS animals received a nonspecific immunoglobulin (rat IgG) intravenously. After 2 hours of observation, intestinal microcirculation was evaluated by using IVM; the plasma levels of monocyte chemoattractant protein-1 (MCP-1) and tumor necrosis factor-alpha (TNF-α) were determined; and LOX-1 expression was quantified in intestinal tissue with Western blot and reverse-transcription polymerase chain reaction (PCR).

**Results:**

LOX-1 inhibition significantly reduced LPS-induced leukocyte adhesion in intestinal submucosal venules (*P *< 0.05). At the protein and mRNA levels, LOX-1 expression was significantly increased in untreated LPS animals (*P *< 0.05), whereas in animals treated with LOX-1 antibody, expression of LOX-1 was reduced (*P *< 0.05). MCP-1 plasma level was reduced after LOX-1 antibody administration.

**Conclusions:**

Inhibition of LOX-1 reduced leukocyte activation in experimental endotoxemia. LOX-1 represents a novel target for the modulation of the inflammatory response within the microcirculation in sepsis.

## Introduction

Sepsis, severe sepsis, and septic shock are attributed with a high incidence and mortality in critically ill patients [1]. The development of septic multiple organ failure is linked to the impairment of the microcirculation of vital and nonvital organs. Several factors contribute to the impairment of the microcirculation in sepsis, including disseminated intravascular coagulation, capillary leakage, and leukocyte adhesion and infiltration [2].

LOX-1 is a 50-kDa type II membrane protein that structurally belongs to the C-type lectin family, with a short intracellular N-terminal hydrophilic and a long extracellular C-terminal hydrophilic domain separated by a hydrophobic domain of 26 amino acids [3]. Information concerning the pathophysiologic role of LOX-1 is accumulating. The unique lectin-like structure enables LOX-1 to recognize a wide range of negatively charged substances, including oxidized low-density lipoproteins (OxLDLs), damaged or apoptotic cells, (endo)toxins, and pathogenic microorganisms [3]. After binding to LOX-1, these ligands can either be internalized by endocytosis or phagocytosis or can remain at the cell surface for adhesion. Under physiologic conditions, LOX-1 may serve to clean up cellular debris and other related materials, and it might play a role in host defense [4-6]. In pathologic states, LOX-1 might be involved in the binding of OxLDL and cellular ligands to activate endothelial cells, the transformation of smooth muscle cells (SMCs), and the accumulation of lipids in macrophages, especially important in the development of atherosclerosis [7-9]. The expression of LOX-1 is induced by stimuli as rapidly as other kinds of cell-adhesion molecules and selectins, suggesting that LOX-1 belongs to the so-called class of immediate-early genes [10]. LOX-1 is a potent mediator of ''endothelial dysfunction'': binding of endothelial LOX-1 by ligands induces superoxide generation, inhibits nitric oxide production, enhances endothelial adhesiveness for leukocytes, and induces expression of chemokines [11-13].

In a rat model with endotoxin-induced uveitis, an antibody against LOX-1 suppressed leukocyte infiltration and protein exudation [10]. However, the effects of LOX-1 inhibition on leukocyte activation during systemic inflammation must be further elucidated.

The intestinal microcirculation is crucial in the pathogenesis of septic multiple organ failure [2]. Therefore, the aim of our experimental study was to evaluate the effects of LOX-1 inhibition on endotoxin-induced leukocyte adherence and the impaired capillary perfusion in the intestinal microcirculation during experimental endotoxemia by using intravital microscopy (IVM).

## Materials and methods

### Animals

The study was performed in accordance with internationally recognized guidelines, the local Instructions for Animal Care of the University of Greifswald, and the German Law on the Protection of Animals (approved by the Landesamt für Landwirtschaft, Lebensmittelsicherheit und Fischerei Mecklenburg-Vorpommern). Forty male Lewis rats (200 to 250 g) were obtained from Charles River Laboratories (Sulzfeld, Germany) and kept under constant conditions of a 12-hour light/dark cycle at 25°C with a humidity of 55%. After the experiments, the animals were sacrificed by using a pentobarbital overdose.

### Anesthesia and preparation

Anesthesia was induced by intraperitoneal injection of a bolus of 60-mg/kg pentobarbital (Synopharm GmbH & Co. KG, Barsbüttel, Germany). To maintain an adequate depth of anesthesia, the animals received 5 mg/kg pentobarbital intravenously every hour. For preparation, the animals were placed in a supine position, and a straight skin incision from the chin to the sternum was made. The polyethylene catheters (PE 50; internal diameter, 0.58 mm; external diameter, 0.96 mm; Portex; Smiths Medical, Hythe, Kent, UK) were introduced into the left external jugular vein and common carotid artery. The intraarterial catheter provided a continuous monitoring of mean arterial blood pressure (MAP) and heart rate (HR) (monitor: Philips LDH 2106/00; Philips, Eindhoven, The Netherlands). To secure the airway, a trimmed venous catheter (16 G, BD Insyte-W; Becton Dickinson GmbH, Germany) was introduced into the trachea via tracheotomy. The animals breathed spontaneously in room air. To maintain a constant body temperature of 37°C ± 0.5°C, the animals were placed on an electric blanket. To expose the intestine, a median laparotomy subsequently was performed from the xyphoid to the symphysis.

### Protocol

We administrated 5 mg/kg lipopolysaccharide (LPS) from *Escherichia coli*, serotype O157:H7 (Sigma-Aldrich Chemie, Steinheim, Germany) intravenously in 30 animals. Fifteen minutes after LPS administration, 10 of the animals received 10 mg/kg LOX-1 antibody (LPS/Anti-LOX group) intravenously. To differentiate specific LOX-1 effects from unspecific antibody effects, another 10 animals received rat immunoglobulin G (LPS/IgG group). The remaining 10 animals did not receive any treatment (LPS group). The control group (CON) animals received an equivalent volume of placebo (normal saline; Delta Select GmbH, Dreieich, Germany).

Intravital microscopy was performed 2 hours after LPS administration. Blood samples for the laboratory analyses were drawn 30 and 120 minutes after the start of the experiments. At the end of the IVM experiments, animals were sacrificed, and samples of intestinal tissue taken for further protein and mRNA analysis.

### Intravital fluorescence microscopy

A part of the intestine approximately 5 cm proximal to the ileocecal valve was identified and placed on an adjustable object table on the microscope. The configuration and procedure for IVM were described previously [14]. In brief, leukocytes were stained *in vivo *by an intravenous injection of 0.2 ml 0.05% Rhodamine 6G solution (Sigma-Aldrich Chemie GmbH, Steinheim, Germany). Capillary perfusion was made visible by the administration of 5% FITC-albumin solution (1 ml/kg, intravenous; Sigma-Aldrich Chemie). For evaluation of the leukocyte adhesion, the intestinal section was focused at the submucosal level. Six visual fields containing nonbranching, collecting venules (V1) over a length of at least 300 μm, as well as another six visual fields revealing similar postcapillary venules (V3), were observed and recorded for 35 seconds each. To obtain comparable results, we selected vessels of comparable size (V1, 60 to 80 μm; V3, 30 to 40 μm). The same procedure was done by focusing random fields of the capillaries within the longitudinal as well as the circular muscle layer and the mucosa. Evaluation of all the video sequences was accomplished after the experiments by analyzing the videotapes off line with a computer-connected video system and software (CapImage; Zeintl, Heidelberg, Germany). Leukocyte adherence was defined as the number of leukocytes that stayed immobile for at least 30 seconds on an oblique, cylindrical endothelial surface (number/mm^2^). FCD was measured as the length of capillaries with observable erythrocyte perfusion in relation to a predetermined rectangular field (cm/cm^2^).

### Cloning of rat *LOX-1 *gene and preparation of polyclonal antibodies

The coding region for the triple-repeat motive comprising amino acids 94 to 232 of LOX-1 protein from rat was amplified with the oligonucleotides 5'-CGGGATCCAAGAATCAAAGAGGGAACTGAA-3' (5'-end) and 5'-CCGCTCGAGACCTGAAGAGTTTGCAGCTCT-3' (3'-end), which introduced *Bam*HI and *Xho*I restriction sites (underlined). The *Bam*HI/*Xho*I-fragment was then fused in frame to the *glutathion-S-transferase *(GST) gene into pGEX-5X-3 (GE Healthcare, Freiburg, Germany) to obtain the plasmid pGEX-5X-3/AA94-232. The construct was sequenced, and identity with the published *LOX-1 *sequence from rat was confirmed. Recombinant GST/AA94-232 protein was produced in *Escherichia coli *strain BL21(DE3)pLysS, purified, and cleaved with Factor Xa for 16 hours. AA9494-232 was used to prepare a polyclonal antiserum in rabbits with a standard immunization protocol [15].

### Quantitative reverse transcription polymerase chain reaction

Quantification of *LOX-1 *and *β-actin*, as an endogenous housekeeping gene, mRNA expression was performed by using mRNA Assays-on-Demand (Applera Deutschland GmbH, Darmstadt, Germany) on an Applied Biosystems ABI Prism 7700, as described previously [16].

### Protein isolation and quantification

At the end of the IVM experiments, animals were sacrificed, and intestinal tissue was dissected, washed twice with media, and homogenized in 10 m*M *Tris (pH 7.4, 1 m*M *sodium ortho-vanadate, and 1% (wt/vol) SDS). Protein concentrations were measured by using the bicinchoninic acid (BCA) Protein Assay Kit (Perbio Science, Bonn, Germany).

### Laboratory analyses

Blood samples were drawn 30 and 120 minutes after start of the experiments. Monocyte chemoattractant protein (MCP)-1 and tumor necrosis factor-alpha (TNF-α) plasma levels were measured according to the manufacturer's instructions (FlowCytomix; Bender MedSystems, Vienna, Austria).

### Statistical analyses

Results were analyzed by using the software Prism 5 (GraphPad Software, La Jolla, CA, USA). First, data were tested for normal distribution by using the Kolmogorov-Smirnov test. If normal distribution was established, one-way analysis of variance (ANOVA) was performed. If significant differences appeared, a *post hoc *analysis with Dunn's Multiple Comparison Test was conducted. The investigations of values in multifactorial design were examined by means of two-way analysis of variance (two-way repeated-measures ANOVA). A value of *P *< 0.05 was considered statistically significant.

## Results

The protocol was performed as outlined. All animals survived the observation period and could be included in the study.

### Microcirculation

**Figure 1:**
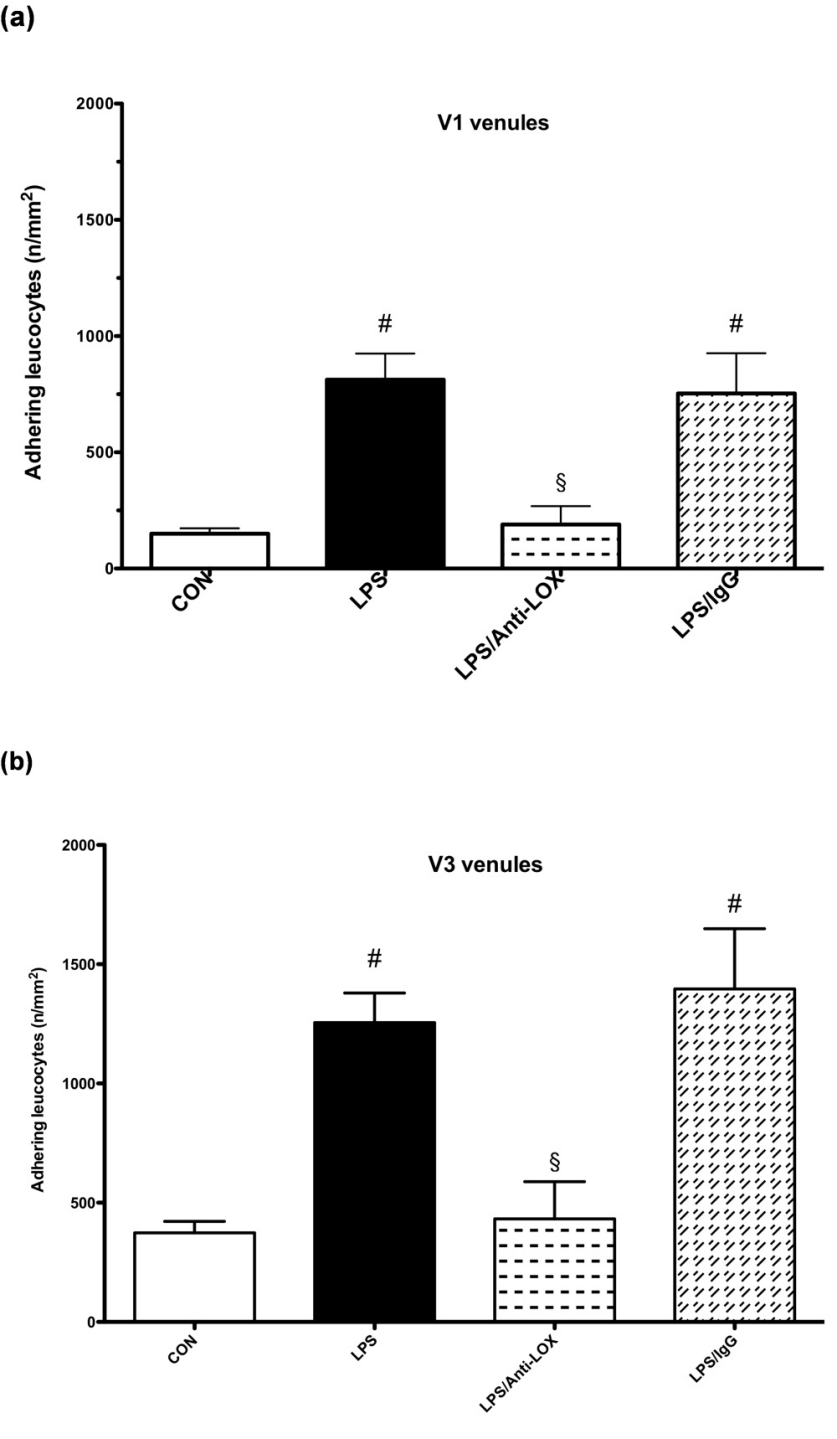
**Adherent leukocytes in V1 (a) and V3 (b) venules (*n*/mm^2^)**. CON, control group (*n *= 10); LPS, lipopolysaccharide group (*n *= 10); LPS/Anti-LOX, lipopolysaccharide and LOX-1-antibody group (*n *= 10); LPS/IgG, lipopolysaccharide and unspecific immunoglobulin group (*n *= 10). #*P *< 0.05 vs. CON; §*P *< 0.05 vs. LPS.

We observed a significant increase of the number of adherent leukocytes in V1 (collecting) and V3 (postcapillary) venules of untreated LPS animals compared with control animals (Figure 1a and 1b; *P *< 0.05). This increase was completely abolished in the LOX-1-antibody-treated LPS group (*P *< 0.05). Unspecific immunoglobulin administration did not influence leukocyte adhesion in LPS-challenged animals. Functional capillary density was not significantly impaired in these endotoxemia experiments (Table 1).

**Table 1:** Functional capillary density.

	CON	LPS	LPS/Anti-LOX	LPS/IgG
Longitudinal muscle layer (mm)	140.5 ± 21.7	160.0 ± 10.9	169.4 ± 10.7	152.2 ± 29.9
Circular muscle layer (mm)	90.7 ± 33.9	115.7 ± 41.8	131.7 ± 21.0	119.8 ± 37.6
Mucosa (mm)	471.2 ± 51.2	456.9 ± 65.4	507.4 ± 51.5	526.9 ± 45.1

CON, control group; LPS, lipopolysaccharide group (untreated); LPS/Anti-LOX, LPS animals treated with the LOX antibody; LPS/IgG, LPS animals treated with unspecific immunoglobulin G; functional capillary density, cm/cm^2^; values expressed as mean ± SD; *n *= 10 per group.

### LOX-1 expression

**Figure 2:**
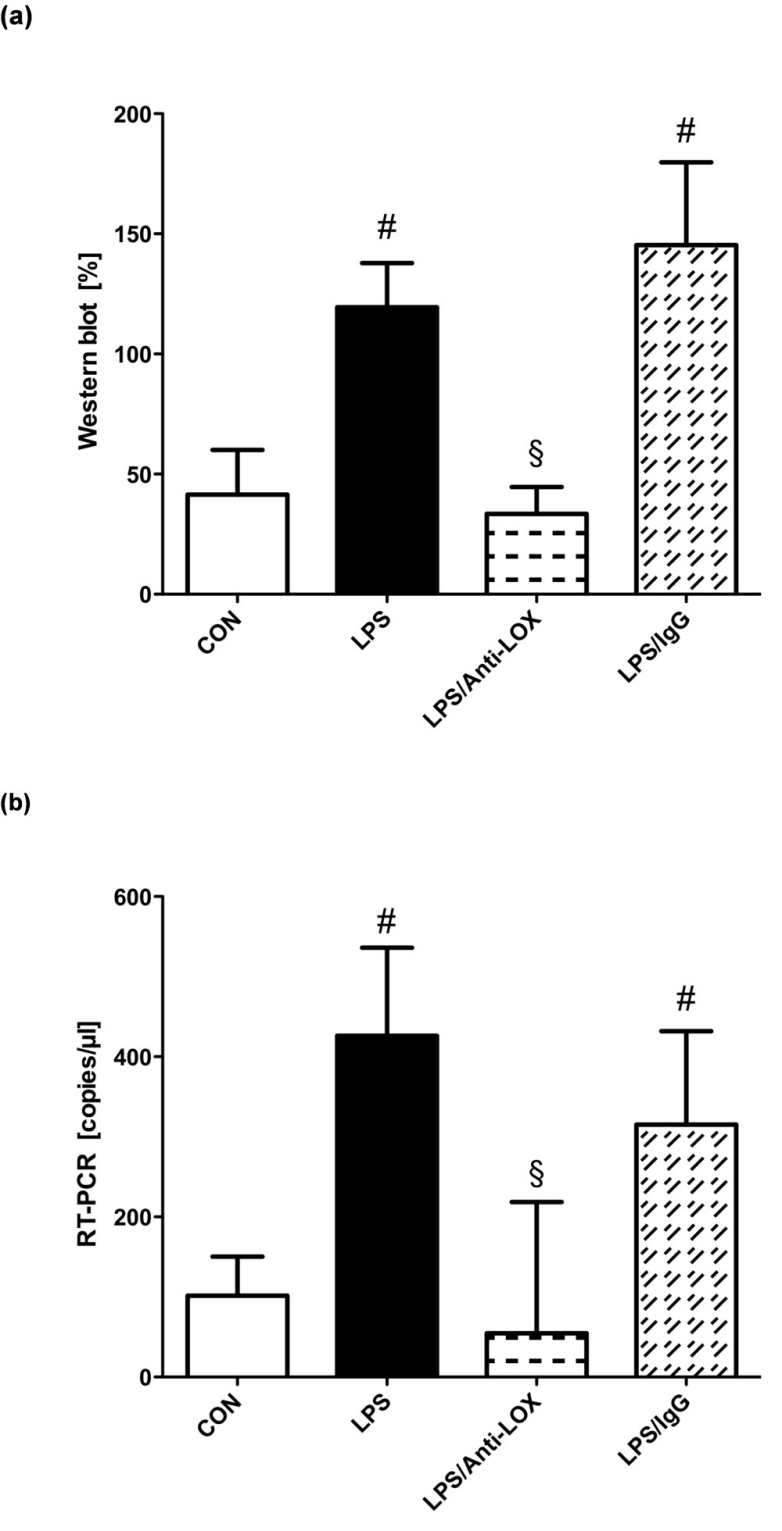
**Expression of LOX-1 protein (a) and mRNA (b) in rat intestine**. Intestinal tissue was harvested from control (CON), lipopolysaccharide (LPS), lipopolysaccharide, LOX-1-antibody treated (LPS/Anti-LOX), and lipopolysaccharide and unspecific immunoglobulin treated (LPS/IgG) animals (*n *= 10 for each group). Total protein and mRNA were extracted from rat intestine tissue, and the expression of LOX-1 was evaluated with Western blot and reverse transcription PCR, respectively. #*P *< 0.05 vs. CON; §*P *< 0.05 vs. LPS.

Endotoxemia resulted in a significant increase in LOX-1 protein expression (Figure 2a). Administration of the antibody against LOX-1 significantly prevented the upregulation of LOX-1 protein expression. Unspecific immunoglobulin had no effect on LOX-1 protein expression in the presence of LPS. Effects of LPS and anti-LOX-1 were confirmed at the mRNA level by RT-PCR (Figure 2b).

### MCP-1 and TNF-α release

**Figure 3:**
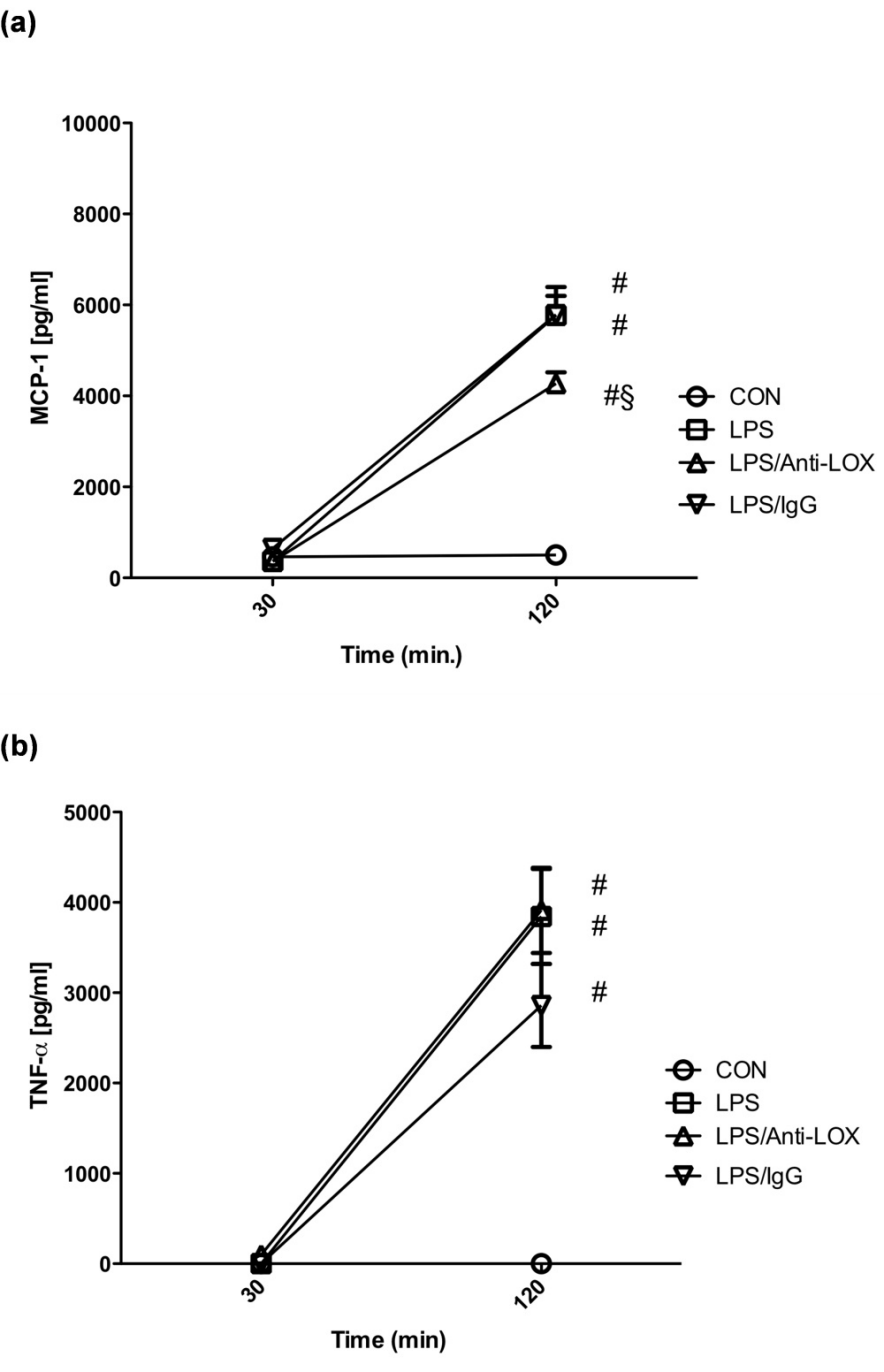
**Plasma levels of MCP-1 (a) and TNF-α (b)**. CON, Control group (*n *= 10); LPS, lipopolysaccharide group (*n *= 10); LPS/Anti-LOX, lipopolysaccharide and LOX-1-antibody group (*n *= 10); LPS/IgG, lipopolysaccharide and unspecific immunoglobulin group (*n *= 10). #*P *< 0.05 vs. CON; §*P *< 0.05 vs. LPS.

MCP-1 plasma levels were significantly elevated in all endotoxemic groups (Figure 3a). MCP-1 release was significantly reduced in the LPS/Anti-LOX group in comparison to untreated or IgG-treated LPS animals. TNF-α concentrations were increased in all endotoxemic animals compared with those in the control group (Figure 3b; *P *< 0.05).

### Macrocirculation

**Figure 4:**
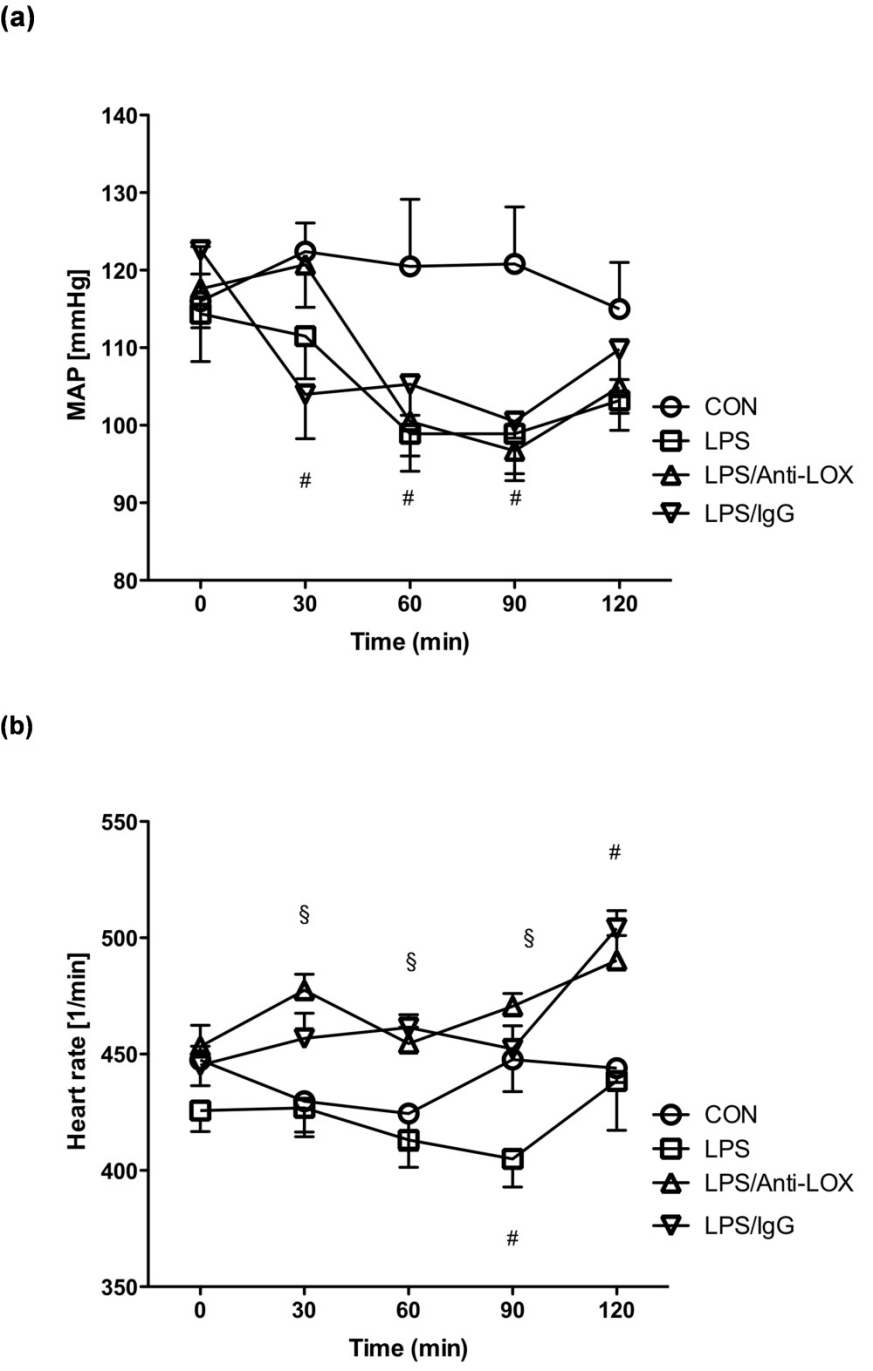
**Mean arterial pressure and heart rate**. CON, Control group (*n *= 10); LPS, lipopolysaccharide group (*n *= 10); LPS/Anti-LOX, lipopolysaccharide and LOX-1-antibody group (*n *= 10); LPS/IgG, lipopolysaccharide and unspecific immunoglobulin group (*n *= 10); #*P *< 0.05 vs. CON; §*P *< 0.05 vs. LPS.

Mean arterial pressure (MAP, Figure 4a) and heart rate (Figure 4b) were stable in control animals over the 2-hour period of the investigation. Between 30 and 90 minutes, a significant decrease of MAP in all endotoxemic groups was noted compared with that in the control group. A significant decrease of heart rate was observed in the LPS-only group at 90 minutes, as compared with the control group. Endotoxemic groups plus treatment (either LOX-1 antibody or unspecific immunoglobulin) showed a significant increase in heart rate between 30 and 90 minutes, which persisted for the duration of the experiments. Heart rates were also significantly higher in these groups as compared with the LPS-only group.

## Discussion

Administration of antibodies against LOX-1 significantly reduced endotoxin-induced leukocyte adherence in intestinal submucosal venules. LOX-1 expression was reduced significantly at both mRNA and protein levels in animals treated with the antibody. MCP-1 plasma levels were found to be decreased after administration of antibodies against LOX-1.

The exposure of LDL to oxidative stress generates OxLDL. The expression of the OxLDL receptor LOX-1 is upregulated by the increased occurrence of OxLDL. Interestingly, the OxLDL-induced upregulation is inhibited by antibodies against LOX-1 [17,18]. Endotoxemia and sepsis are pathologic conditions with increased oxidative stress and release of reactive oxygen species (ROS). Our findings suggest that LOX-1 antibodies are also able to reduce the endotoxin-induced expression of LOX-1.

Because ROS function as signal-transduction molecules that modulate the activity of the transcription factors, various changes via gene expression accompany the changes in redox status of the cells. Activation of NF-κB, a redox-sensitive transcription factor, induces upregulation in the expression of vasoconstrictive molecules, adhesion molecules, and chemokines [19,20]. Actually, activation of LOX-1 in endothelial cells induces the expression of endothelin-1, AT1 receptor, E-selectin, P-selectins, VCAM-1 and ICAM-1 (30), and MCP-1 [11]. These gene products increase vascular tonus and promote leukocyte-endothelial interactions and the release of additional pro-inflammatory signals. We confirmed in our experiments that leukocyte adhesion and MCP-1 levels can be influenced by LOX-1 inhibition in experimental endotoxemia in rats.

TNF-α is an early proinflammatory cytokine. Kume *et al. *[21] showed that TNF-α increases cell-surface expression of LOX-1 in a concentration-dependent manner, and peak levels of LOX-1 expression are at 8 to 12 hours with continuous TNF-α stimulation *in vitro*. TNF-α appeared to activate the transcription of LOX-1, as measured by nuclear run-off assay. Time to peak concentrations of TNF-α has been suggested to be 1 hour after endotoxin challenge [22]. In our experiments, TNF-α was measured about 2 hours after endotoxin challenge. This may explain why we did not observe differences in the TNF-α levels between the experimental groups.

OxLDL itself has also been well known to play a key role in the adherence of monocytes to the activated endothelium. Possible intracellular processes include activation of protein kinase C, mitogen-activated protein kinase (MAPK), and the subsequent upregulation of MCP-1 [12,23,24]. Li *et al. *[11] found that incubation of endothelial cells with Ox-LDL increased the phosphorylation of MAPK. In these experiments, OxLDL also upregulated MCP-1 expression (protein and mRNA) and monocyte adhesion to the endothelial cells through activation of LOX-1. In a model of low-dose endotoxin-induced uveitis, antibodies against LOX-1 efficiently suppressed leukocyte infiltration and protein exudation. *In situ *videomicroscopic analyses of leukocyte interactions with retinal veins revealed that anti-LOX-1 antibody reduced the number of rolling leukocytes and increased the velocity of rolling, suggesting that LOX-1 functions as a vascular tethering ligand. The ability of LOX-1 to capture leukocytes under physiologic shear was confirmed in an *in vitro *flow model [10]. We also were able to show that endotoxin-induced leukocyte adhesion can be influenced by anti-LOX-1 administration. Leukocyte adhesion was completely abolished in the LOX-1 antibody-treated LPS group in rats.

Influences of a reduced perfusion pressure, the typical response to endotoxemia, on the findings within the microcirculation cannot be excluded completely, but the reduction in mean arterial blood pressure in our experiments was only temporary and still in a physiologic range. At the time of the evaluation of the microcirculation, perfusion pressure in endotoxemic animals was not significantly different from that of controls. Furthermore, capillary perfusion, as measured by the functional capillary density, was unchanged in endotoxemic animals, also indicating a negligible impact of the perfusion pressure in our experiments. Several studies observed a significant impairment of functional capillary density during experimental endotoxemia [25-27]. However, the extent of the impairment of the functional density depends on several factors (for example, the serotype, the dosage, and the endotoxin activity of the LPS used for the induction of endotoxemia and the organ/tissue studied for changes in the microcirculation). It was interesting to observe that FCD response and leukocyte activation can be dissociated. We interpret the missing effect of endotoxemia on the FCD in our experimental study as associated with the low severity of the model (no septic shock).

Reduction of leukocyte adhesion and impact on functional capillary density and cytokine response, as well as the effect on survival by administration of LOX-1 antibodies in endotoxemia, should be studied in further animal experiments. These studies will verify the potential use of this therapeutic approach in a clinical setting.

## Conclusions

Inhibition of the lectin-like oxidized low-density lipoprotein receptor-1 resulted in a reduction of endotoxin-induced intestinal leukocyte adhesion. Therefore, this receptor may represent a novel target for the modulation of the inflammatory response within the microcirculation in sepsis.

## Key messages

• Lectin-like oxidized low-density lipoprotein receptor-1 (LOX-1) is involved in leukocyte recruitment.

• Systemic leukocyte activation represents a crucial factor in the pathogenesis of sepsis.

• Inhibition of LOX-1 reduced leukocyte activation in experimental endotoxemia.

• In rats treated with LOX-1 antibody, expression of LOX-1 was reduced.

• LOX-1 represents a novel target for the modulation of the inflammatory response within the microcirculation in sepsis.

## Abbreviations

ELISA: enzyme-linked immunosorbent assay; FCD: functional capillary density; FITC: fluorescein isothiocyanate; HR: heart rate; IgG: immunoglobulin G; IL: interleukin; IVM: intravital fluorescence microscopy; LDL: low-density lipoprotein; LOX-1: lectin-like oxidized low-density lipoprotein receptor-1; LPS: lipopolysaccharide; MAP: mean arterial pressure; MCP-1: monocyte chemoattractant protein-1; OxLDL: oxidized low-density lipoprotein; ROS: reactive oxygen species; RT-PCR: reverse transcription polymerase chain reaction; SMC: smooth muscle cell; TNF-α: tumor necrosis factor-alpha; V1: collecting venule; V3: postcapillary venule.

## Competing interests

Parts of this work were supported by a grant from the Department of Cardiovascular Medicine within the NBL3 program (reference 01 ZZ 0403) of the German Federal Ministry of Education and Research (to ML and SBF). Supported in part by Research project MZO 00179906 from the University Hospital Hradec Kralove, Czech Republic (to VC).

## Authors' contributions

ML and MO performed cloning, expression, and antibody preparation of LOX-1, Western blot, and RT-PCR analysis. SW and CT carried out intravital microscopy. ML and CL conceived of the study, analyzed data, and drafted the manuscript. DP, MM, and VC made substantial contributions to the conception and design of the study, and DP supervised the IVM experimental procedure. SBF, JZ, SW, and OH have been involved in revising the manuscript critically for important intellectual content. All authors read and approved the final manuscript.
